# Solitary Osteochondroma of the Tibial Tuberosity Mimicking Osgood-Schlatter Lesion: A Rare Cause of Anterior Knee Pain in Adolescents: A Case Report

**DOI:** 10.5704/MOJ.1607.009

**Published:** 2016-07

**Authors:** G Balaji, P Palaniappan, S Nema, J Menon

**Affiliations:** Jawaharlal Institute of Postgraduate Medical Education and Research (JIPMER), Pondicherry, India

**Keywords:** Knee tibia, exostoses, osteochondroma, adolescent

## Abstract

Osteochondroma arising from the tibial tuberosity is very rare. We report such a case which mimicked OsgoodSchlatter’s disease in an adolescent. A 12 years-old boy presented with swelling over his right proximal tibia of one year duration associated with pain in the last three months. Examination revealed a 4 x 2cm bony mass arising from the proximal tibia. Radiographs revealed an osteochondroma of the tibial tuberosity. Computer tomography and magnetic resonance imaging confirmed the continuity of the medulla of the bony mass to that of the parent bone. Excision biopsy was done. At the final follow up, he was asymptomatic and returned back to his daily activities. We present this case for its rarity, challenges involved in diagnosis and the difficulties encountered in planning the surgery because of involvement of the apophysis and extensor mechanism attachment in a skeletally immature boy.

## Introduction

Osteochondroma is the most common benign bone tumour. It accounts for 10-15% of all bone tumours and around 30% of all benign bone tumours^1^. We report a rare case of osteochondroma arising from the tibial tubercle in an adolescent who presented with anterior knee pain. The challenges in diagnosis and treatment options are discussed.

## Case Report

A 12 years-old boy presented to our outpatient department with swelling and pain over his right knee. The swelling was present for one year, insidious in onset and gradually progressive. The onset of pain was also insidious beginning three months prior to presentation. Pain was aggravated on prolonged walking, playing, sitting cross legged and squatting. There was no preceding history of significant trauma or fever.

On examination, there was a 4 x 2cm globular swelling arising from the anterior aspect of his proximal tibia. The swelling was hard in consistency and non tender. The range of movement of the knee was full but painful on extension and terminal flexion. There were no distal neurovascular deficits.

**Fig. 1:**
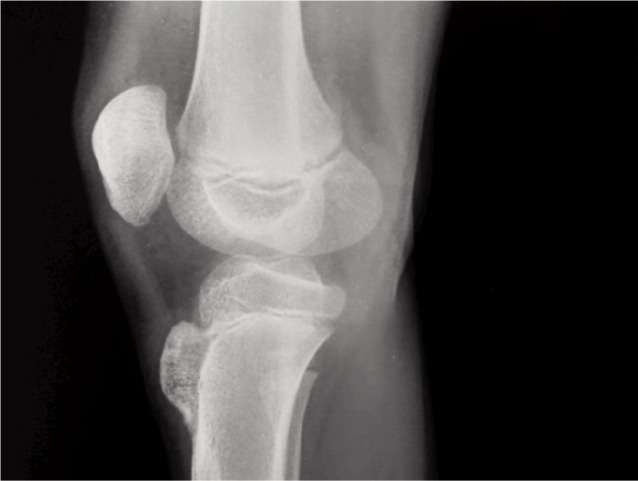
Radiograph of the right knee lateral view shows a bony mass arising from the tibial tuberosity.

**Fig. 2:**
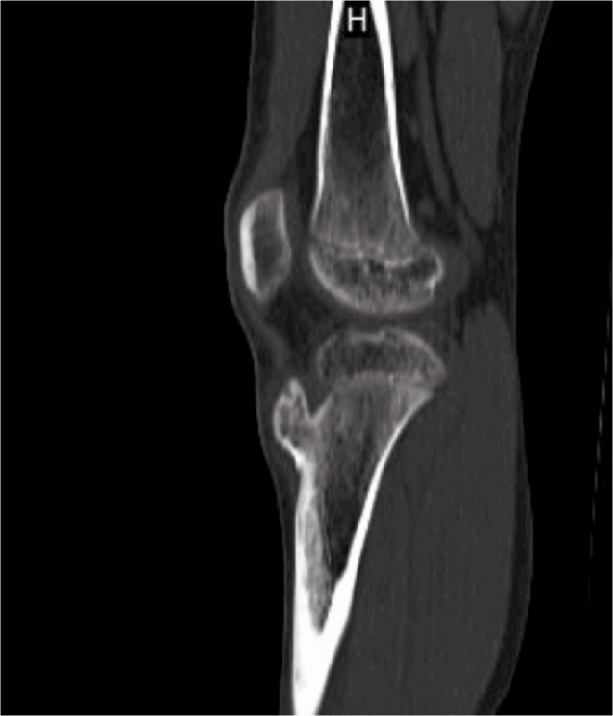
Noncontrast sagittal computer tomography image shows bony excrescence arising from the tibial tubercle with cortical and medullary continuity with the underlying bone.

**Fig. 3:**
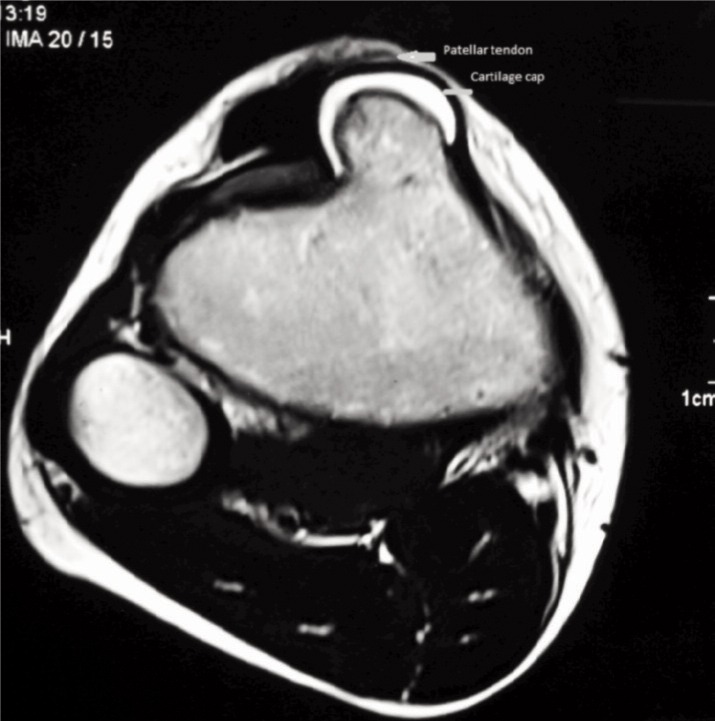
Fat-saturated T2 axial section of magnetic resonance imaging shows a well-defined osseous protrusion from the anteromedial aspect of tibial metaphysis underneath the patellar tendon.

Considering the age of the patient and the location of the mass, clinically we suspected apophysitis of the tibial tuberosity. Blood investigations were normal except alkaline phosphatase which was 713 IU/L (Normal range: 20 - 140 IU/L). Radiograph of the knee revealed a bony outgrowth from the anterior aspect of the tibial tuberosity without any fragmentation suggesting probably an osteochondroma (Fig. 1). Computer tomography (CT) showed 3 x 1.8cm bony projection from the anterior aspect over the tibial tuberosity. The cortex and medulla of the bony projection were continuous with the underlying bone and was pointing cranially (Fig. 2). Magnetic resonance (MR) imaging of the knee showed a well defined broad-based, eccentric, osseous protrusion arising from proximal tibial metaphysis and continuous with the parent bone cortex and trabeculae (Fig. 3). The lesion had a cartilage cap thickness of 2.5mm with no evidence of calcification. The adjacent soft tissues were normal.

**Fig. 4:**
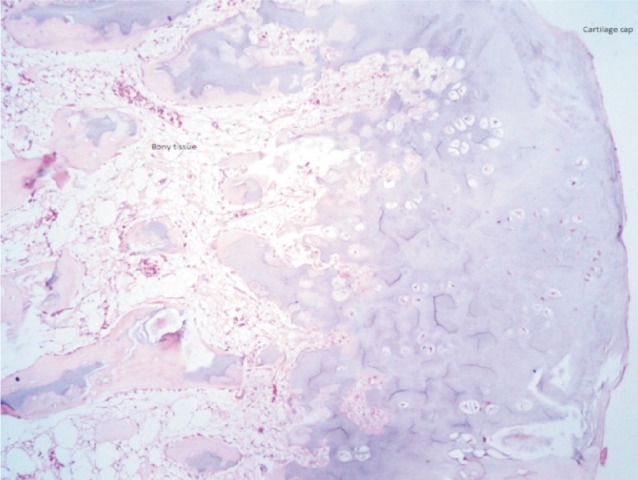
Histopathology picture with hematoxylin and eosin staining shows osseous tissue with cartilage cap suggestive of osteochondroma.

Since he was symptomatic, we planned for an excision biopsy of the mass. Under general anaesthesia, an anteromedial incision was made over the infrapatellar region extending up to the tibial tuberosity. The patellar tendon was retracted laterally. The mass was seen projecting more on the anteromedial aspect. The bony projection was excised *in toto* along with the periosteum. The limb was immobilised for two weeks after which knee mobilisation was started. The histopathological report of the excised globular mass measuring 4 x 3 x 3cm with cartilage cap thickness of 0.2cm was suggestive of osteochondroma (Fig. 4). At the end of final follow up, there was no evidence of pain or recurrence of the swelling. He returned to his routine activities of daily living including playing without any symptoms.

## Discussion

Osteochondromas are usually asymptomatic and are identified incidentally. The symptoms occur following sudden increase in size, secondary to trauma, fracture of the stalk, irritation to the nearby soft tissues causing bursitis, neurovascular impingement and malignant degeneration. Our patient was symptomatic probably because of the unusual location of the lesion beneath the extensor mechanism causing repeated stress on knee movements.

The incidence of osteochondromas arising from the proximal tibia is 15-20%^2^. Osteochondroma arising from tibial tuberosity *per se* is very rare and is not reported in English literature to the best of our knowledge. Clinically, osteochondromas present as painless bony hard swellings arising from the underlying bone. Our patient presented with anterior knee pain. He had pain in the knee both in flexion and extension. This is probably because of the osteochondroma lying deep to the patellar tendon. Every time the knee flexes, the impingement on the patellar tendon by the osteochondroma and also the additional excursion required for the patellar tendon due to the stretching of the tendon over the swelling resulted in pain. The pain on extension could be because of the osteochondroma being located at the attachment of the extensor mechanism resulting in repeated stress.

Our patient presented with a swelling from the tibial tuberosity closely mimicking Osgood-Schlatter’s disease (OSD). It is necessary to clinically differentiate between the two conditions as both are common in adolescents. OSD is due to traction apophysitis of the tibial tubercle, and usually presents with soft tissue swelling near the tibial tubercle and pain is mostly during extension of the knee^3^. Our patient had bony swelling which was painful on both knee flexion and extension. OSD may sometimes present bilaterally which is not common with osteochondroma though they can be present at different sites as in hereditary multiple osteochondromatosis. The other condition to be kept in mind in differential diagnosis is hemimelic epiphyseal dysplasia, also known as Trevor’s disease. This condition is due to a rare developmental disorder as a result of asymmetric epiphyseal cartilage growth^4^ commonly seen in adolescents and presents as a painless bony mass around the joint.

Radiographs in osteochondroma usually show a bony projection arising from the underlying bone. In OSD, there is usually fragmentation of the tibial tubercle whereas Trevor’s disease there are irregular multiple opacities in the epiphysis. CT and MR imaging help in identifying the normal and the abnormal bone, the soft tissue involvement and to determine the continuity of the abnormal mass with that of the medullary cavity of the parent bone.

Decision making on whether to operate or to treat them nonoperatively is very important. The issues on surgical intervention in skeletally immature patients are damage to the physis as a result of extensive excision leading to limb length discrepancy. Ideally, excision of osteochondroma is done after skeletal maturity, so that the mass is usually away from the growth plate thereby reducing the risk of physeal damage.

We decided to operate in this case as our patient had persistent pain affecting his daily routine. The dilemmas we faced before operating were whether to approach the lesion through transtendon or parapatellar approach. What do we do regarding the attachment of the extensor apparatus if the lesion is involving the entire tibial tubercle? How far should we resect the lesion as it is very close to the proximal physis? We decided to approach the mass through a medial parapatellar approach as the mass was more anteromedially based on the imaging. The patellar tendon was retracted laterally and the mass was resected in toto just distal to the level of the physis. We would have had difficulty in excising the medial aspect of the mass if we had used tendon splitting approach. Since the mass was just proximal to the distalmost attachment of the patellar tendon, we were able to preserve the extensor tendon attachment in this particular case. Had it involved the entire distal attachment of extensor apparatus, it would be better to excise the whole mass and attach the tendon to the bone using suture anchors or by attaching the tendon to the bone through a tunnel using non-absorbable sutures by Krakow stitch on the tendon. Though we have excised the entire mass just adjacent to the physis, long term follow up is necessary till skeletal maturity to watch for recurrence or growth disturbance.

In conclusion, clinicians should be aware of this rare entity of osteochondroma of the tibial tubercle, its clinical presentation, differential diagnoses and management difficulties. Adequate imaging is necessary particularly when the patient is symptomatic, to arrive at an early diagnosis. Proper preoperative planning is required along with early intervention so that adolescents will be able to resume their regular day-to-day activities soon after surgery.

**Conflict of interest:** The authors declare that there is no conflict of interest.

## References

[1] Kitsoulis P, Galani V, Stefanak K, Paraskevas G, Karatzias G, Agnantis NJ (2008). Osteochondromas: review of the clinical, radiological and pathological features. In Vivo.

[2] Murphey MD, Choi JJ, Kransdorf MJ, Flemming DJ, Gannon FH (2000). Imaging of Osteochondroma: Variants and Complications with Radiologic-Pathologic Correlation. Radiographics.

[3] Hanada M, Koyama H, Takahashi M, Matsuyama Y (2012). Relationship between the clinical findings and radiographic severity in Osgood–Schlatter disease. Open Access J Sports Med.

[4] Baumfeld DS, Pires R, Macedo BD, Abreu-e-Silva G, Alves TA, Raduan FC (2014). Trevor disease (Hemimelic epiphyseal displasia): 12-year follow-up case report and literature review. Annals Med Health Sci Res.

